# Supplementary material for: Identification of candidate cancer drivers by integrative Epi-DNA and Gene Expression (iEDGE) data analysis
Source: Sci Rep. 2019 Nov 15;9:16904. doi: 10.1038/s41598-019-52886-z (PMC6858347; doi:10.1038/s41598-019-52886-z)
Supplement: Supplementary file 1 — Supplementary Documentation [file 41598_2019_52886_MOESM1_ESM.pdf]

Identification of candidate cancer drivers by integrative Epi-DNA and Gene Expression (iEDGE) data analysis

Amy Li (ajli@bu.edu)[1,2], Bjoern Chapuy (bjoern.chapuy@med.uni-goettingen.de)[3,4], Xaralabos Varelas (xvarelas@bu.edu)[5], Paola Sebastiani (sebas@bu.edu)[2,6], Stefano Monti (smonti@bu.edu)[1,2]

[1]Division of Computational Biomedicine, Boston University School of Medicine, Boston, MA 02118, USA

[2]Bioinformatics Program, Boston University, Boston, MA 02215, USA

[3]Dana-Farber Cancer Institute, Department of Medical Oncology, Brookline, MA 02215, USA

[4]University Medical Center Göttingen, 37075 Göttingen, Germany

[5]Department of Biochemistry, Boston University School of Medicine, Boston, MA 02118, USA

[6]Department of Biostatistics, Boston University School of Public Health, Boston, MA 02118, USA

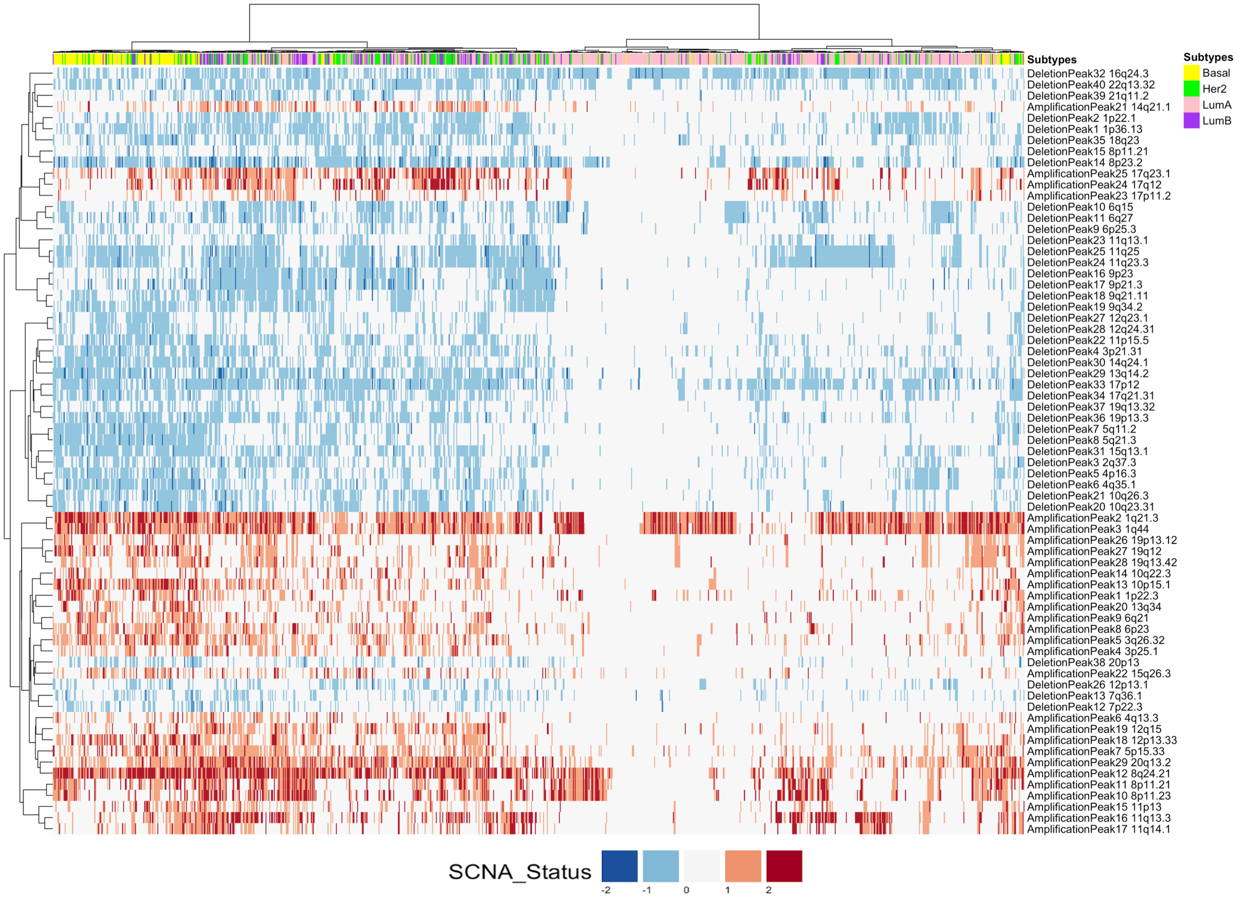

Figure S1. Hierarchical clustering of somatic copy number alteration status (SCNA) across subtyped TCGA breast cancer samples. SCNA_Status legend key: -2 (high-level loss, possibly homozygous deletion): $t > -0.9$; -1 (low-level loss, possibly heterozygous deletion): $-0.9 \leq t < -0.1$; 0 (normal, diploid): $-0.1 \leq t < 0.1$; 1 (low-level amplification): $0.1 \geq t > 0.9$; 2 (high-level amplification): $t > 0.9$

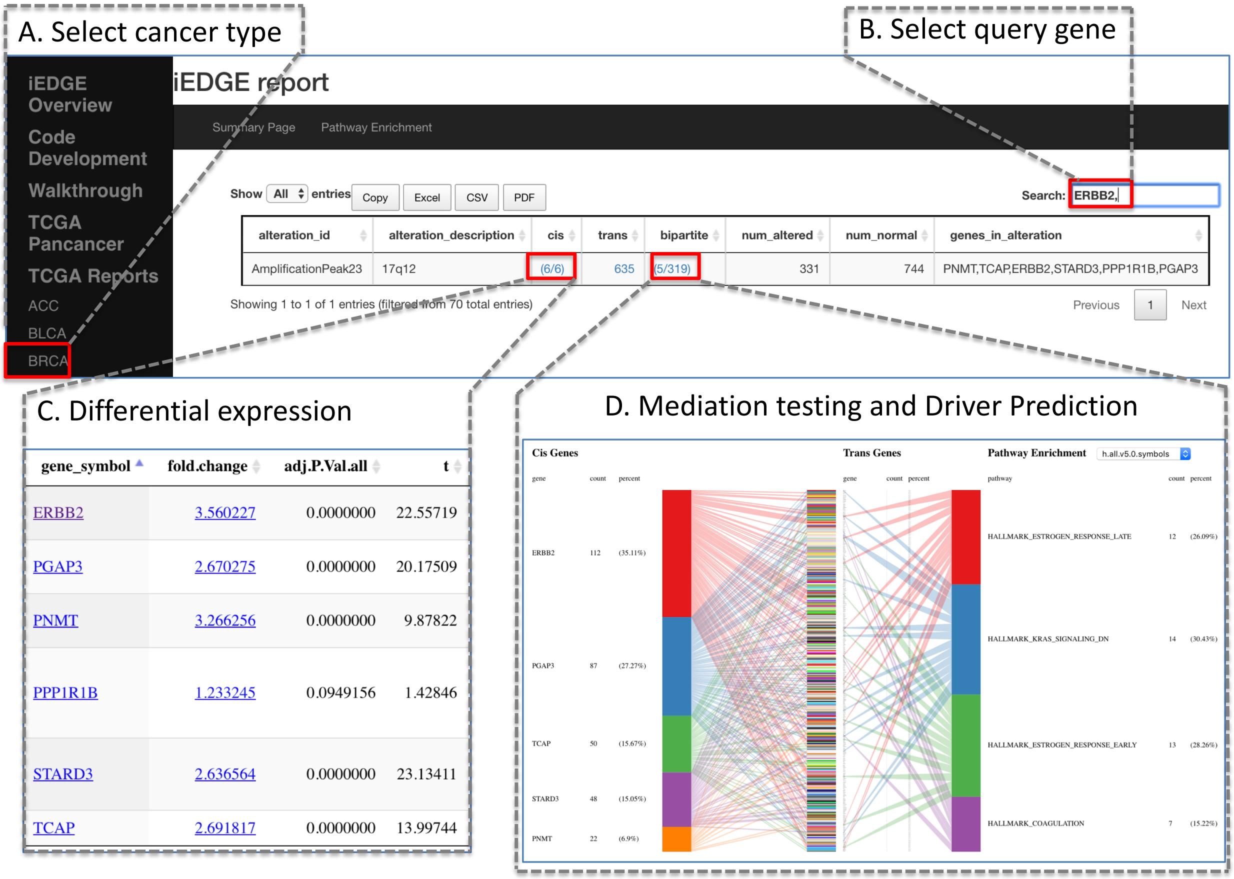

Figure S2. iEDGE web portal overview
(A) Selection of iEDGE report by cancer type
(B) Selection of query gene
(C) Differential expression table report for cis genes
(D) Graphical report of mediation testing and driver prediction
